# Learning the Ropes of Platelet Count Regulation: Inherited Thrombocytopenias

**DOI:** 10.3390/jcm10030533

**Published:** 2021-02-02

**Authors:** Loredana Bury, Emanuela Falcinelli, Paolo Gresele

**Affiliations:** Department of Medicine and Surgery, Section of Internal and Cardiovascular Medicine, University of Perugia, Centro Didattico, Edificio B Piano 1, 06132 Perugia, Italy; loredana.bury@unipg.it (L.B.); emanuelafalcinelli@gmail.com (E.F.)

**Keywords:** inherited thrombocytopenias, platelets, bleeding

## Abstract

Inherited thrombocytopenias (IT) are a group of hereditary disorders characterized by a reduced platelet count sometimes associated with abnormal platelet function, which can lead to bleeding but also to syndromic manifestations and predispositions to other disorders. Currently at least 41 disorders caused by mutations in 42 different genes have been described. The pathogenic mechanisms of many forms of IT have been identified as well as the gene variants implicated in megakaryocyte maturation or platelet formation and clearance, while for several of them the pathogenic mechanism is still unknown. A range of therapeutic approaches are now available to improve survival and quality of life of patients with IT; it is thus important to recognize an IT and establish a precise diagnosis. ITs may be difficult to diagnose and an initial accurate clinical evaluation is mandatory. A combination of clinical and traditional laboratory approaches together with advanced sequencing techniques provide the highest rate of diagnostic success. Despite advancement in the diagnosis of IT, around 50% of patients still do not receive a diagnosis, therefore further research in the field of ITs is warranted to further improve patient care.

## 1. Introduction

Platelets, or thrombocytes, are small and anuclear blood cells with discoid shape and a size of 1.5–3.0 µm which play a crucial function in primary hemostasis. Their normal life span is 9–10 days and total circulating mass 10^12^, thus about 10^11^ platelets are released each day from their bone marrow precursors, megakaryocytes, to maintain a normal circulating platelet count of 1.5 to 4 × 10^9^/L.

Inherited thrombocytopenias (ITs) are a heterogeneous group of congenital disorders characterized by a reduction of platelet number, a widely-variable bleeding diathesis, sometimes aggravated by associated impairment of platelet function, and frequently associated with additional defects, which may heavily impact patient lives.

ITs are rare diseases, with an estimated prevalence of 2.7 in 100,000 [1] although this figure is probably underestimated because they are often misdiagnosed as immune thrombocytopenia (ITP). A recent study on the assessment of the frequency of naturally occurring loss-of-function variants in genes associated with platelet disorders (52% of which were associated with ITs) from a large genome aggregation database showed that 0.329% of subjects in the general population have a clinically meaningful loss-of-function variant in a platelet-associated gene [2].

The first IT, Bernard Soulier syndrome, was described in 1948 and subsequently only few additional forms were reported until Sanger sequencing first, and next generation sequencing later became widely applied rapidly bringing the known ITs from less than a dozen to currently at least 41 disorders caused by mutations in 42 different genes [3,4].

Despite these advancements however, it is estimated that genetic etiology of nearly 50% of patients with IT still remains undefined [5].

## 2. Megakaryocytopoiesis and Platelet Production

Platelets are produced by megakaryocytes (MKs), their giant bone marrow polyploid precursors, through a complex and highly regulated process. During maturation megakaryocytes become polyploid and accumulate massive amounts of proteins and membranes. Then, through a cytoskeletal-driven process, they extend long branching protrusions called proplatelets into sinusoidal blood vessels to release platelets [6]. However, under special conditions associated with strongly increased platelet turnover, platelets can be released through megakaryocyte rupture [7]. More recently, based on the calculation of the rate of proplatelet formation required for physiological platelet replacement, it has been suggested that membrane budding, rather than proplatelet formation, supplies the majority of the platelet biomass in vivo [8]. The release of platelets in lungs by megakaryocytes entered in blood and disintegrated by the impact with the pulmonary microcirculation has also been shown, but its relevance for platelet production is a matter of controversy [9].

The process of megakaryopoiesis involves multiple genes, coding for transcription factors, cytoskeletal proteins, membrane receptors and signaling proteins, which regulate megakaryocyte differentiation and platelet formation and release. Variants in any of these genes may cause IT.

## 3. Hereditary Disorders of Platelet Number

Given the wide heterogeneity of IT, there is no consensus on their classification, and several criteria have been proposed, such as on clinical features (e.g., age at presentation, severity, associated developmental abnormalities), platelet size or inheritance pattern (e.g., autosomal dominant, autosomal recessive and X-linked) [4,10,11].

Here we have grouped them according to the pathogenic mechanisms of thrombocytopenia.

**Figure 1:**
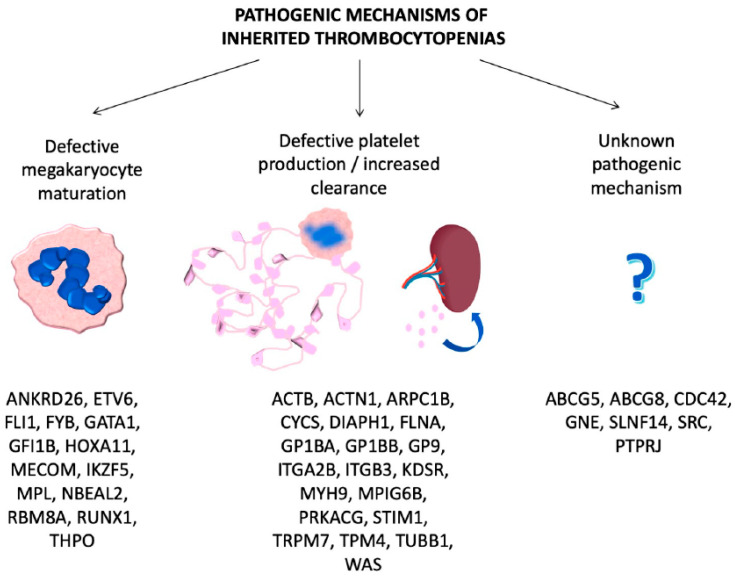
Genes involved in inherited thrombocytopenias classified according to the pathogenic mechanisms.

ITs are primarily caused by mutations in genes involved in megakaryocyte differentiation, maturation and platelet production [12] (Table 1, Figure 1).

### 3.1. ITs Caused by Defective Megakaryocyte Maturation and Differentiation

ITs due to defective differentiation of hematopoietic stem cells (HSCs) into MKs are characterized by the absence or severe reduction in the number of bone marrow MKs. 

ITs caused by altered MK maturation are characterized by a normal or increased number of bone marrow MKs which however are immature, dysmorphic and dysfunctional and include at least 14 different forms. Eight of these are caused by mutations of transcription factors with a key role in megakaryopoiesis, i.e., RUNX1, FLI1, GATA1, GFI1b, ETV6, HOXA11, MECOM, IKZF5. These transcription factors regulate, as activator or repressor, the expression of numerous genes, therefore these disorders are characterized by the concurrent alterations of multiple steps in MK and platelet development. For instance, RUNX1 transactivates transcription factors involved in MK maturation, proteins of the MK cytoskeleton (MYH9, MYL9, MYH10) or implicated in α and dense granule development (RAB1B, PLDN, NFE2) and members of the MK/platelet signaling pathways (ANKRD26, MPL, PRKCQ, ALOX12, PCTP) [17]. FLI1 activates the transcription of several genes associated with the production of mature MKs, including *MPL*, *ITGA2B*, *GP9*, *GPIBA* and *PF4* [52]. Thrombocytopenia of TCPT/JBS, caused by deletions of the long arm of chromosome 11q, is due to reduced expression of *FLI1* which is included in the deleted region.

Disorders caused by GATA1 and GFI1B mutations are associated with erythrocyte abnormalities showing the essential role of these transcription factors in controlling also red cell production. Moreover, the predisposition to haematological neoplasms of patients with *RUNX1* and *ETV6* variants highlights how these pathogenic variants also disrupt the homeostasis of myeloid and multipotent progenitors, respectively. Amegakaryocytic thrombocytopenia with radio-ulnar synostosis (ATRUS), a rare IT which often evolves in trilinear bone marrow failure, is due to variants in HOXA11 and MECOM, members of a family of genes encoding for DNA-binding proteins involved in the regulation of early hematopoiesis [53]. IKZF5 is a transcription factor with a non-clear role in hematopoiesis and is involved in IKZF5-RT [21]. 

Variants in THPO, the gene coding for thrombopoietin, a growth factor essential for hematopoietic stem cell survival and megakaryocyte maturation, and in *MPL*, coding for the thrombopoietin receptor, cause *THPO*-related thrombocytopenia (*THPO*-RT) and congenital amegakaryocytic thrombocytopenia (CAMT), respectively. *FYB*-RT is caused by variants in the FYB gene, coding for a cytoskeletal protein [16], and thrombocytopenia-absent radius is caused by variants in RBM8A, a protein of the exon-junction complex [24]. Finally, ANKRD26 and NBEAL2, proteins with an unknown role, are involved in *ANKRD26*-RT [13] and Gray platelet syndrome (GPS) [23], respectively.

### 3.2. ITs Caused by Defective Platelet Production/Clearance

ITs derived from defects of the generation of proplatelets from mature MKs and/or of the conversion of proplatelets to platelets in the bloodstream are characterized by normal MK differentiation and maturation but by ectopic release of platelets in the bone marrow and/or increased clearance of platelets from the circulation. Most of these forms are associated with enlarged platelets and derive from mutations in genes encoding for components of the acto-myosin or microtubular cytoskeletal system, such as *MYH9*, *ACTN1*, *FLNA*, *TPM4*, *TRPM7* or *TUBB1*, or from mutations of genes for the major membrane glycoprotein (GP) complexes GPIb/IX/V and GPIIb/IIIa that indirectly affect cytoskeletal structure or reorganization, i.e., like biallelic and monoallelic Bernard Soulier syndrome (BSS) and *ITGA2B/ITGB3*-RT. In the latter case macrothrombocytopenia results from the disruption of the interactions of integrins with the actomyosin cytoskeleton which is essential for preserving MK cytoskeletal structure and organization. For instance, *ITGA2B/ITGB3*-RT is due to gain-of-function variants resulting in the constitutive, inappropriate activation of GPIIb/IIIa triggering outside-in signaling with consequent altered remodeling of the actin cytoskeleton [35,54,55]. Another example is platelet type VWD, or pseudo von Willebrand, due to gain-of-function mutations that increase the affinity of GPIbα for VWF with the consequent triggering of the Src kinases pathway downstream of activated GPIbα [34].

Wiskott–Aldrich syndrome (WAS) is a syndromic IT and X-linked thrombocytopenia (XLT) is a milder variant with only isolated thrombocytopenia which derive from mutations in the WAS gene leading to defective expression or activity of its product WASp. WASp is expressed exclusively in hematopoietic cells and has a key role in actin polymerization and cytoskeleton rearrangement. Studies in mice have shown ineffective platelet production with ectopic proplatelet formation (PPF) within the bone marrow and impaired SDF1-driven MK migration to the vascular niche [56]. The observation that splenectomy enhances the platelet count in WAS and XLT patients however, suggests that increased platelet clearance is also an important mechanism of thrombocytopenia in these disorders.

An additional group of IT belonging to those caused by impaired platelet production is due to variants in genes not directly involved in proplatelet formation, such as *CYCS*-RT, caused by dysfunction of a mitochondrial protein that causes thrombocytopenia by enhancing an apoptotic pathway [30], or *PRKACG*-RT, leading to dysfunction of PKA, which activates many proteins involved in megakaryocyte and platelet function such as FLNa and GPIbβ [40]. The Stormorken syndrome is due to gain of function mutations of STIM1 [57]. In these patients platelets circulate in an activated state due to a constitutively active store operated Ca^2+^ release-activated Ca^2+^ (CRAC) channel which triggers Ca^2+^ entry with consequent increased clearance of activated platelets by the spleen which causes a reduction in the number of circulating platelets [58].

Ectopic proplatelet formation in bone marrow is a peculiar mechanism causing thrombocytopenia in *FYB*, *GP1BA* (gain-of-function variants) and *MYH9*. 

### 3.3. ITs Caused by Unknown Pathogenic Mechanisms

One last group of ITs is caused by variants in genes not known to be involved in megakaryocyte maturation or platelet production, and that cause thrombocytopenia by still unknown mechanisms.

An interesting IT is thrombocytopenia associated with sitosterolemia, a rare autosomal recessive disorder caused by mutations in two adjacent ATP-binding cassette transport genes (*ABCG5* and *ABCG8*) encoding proteins (sterolins-1 and -2) that pump sterols out of cells [59]. Among the manifestations of this complex disorder due to the accumulation of sterols in plasma and cell membranes are haematological abnormalities, including thrombocytopenia, provoked by the increased stiffness of sterol-enriched membranes with possible enhanced susceptibility to lysis and rupture [60].

Another recently discovered gene causing IT is *SLFN14*, an endoribonuclease degrading mRNA [49,61,62]. Alongside reduced platelet number, these patients show increased platelet clearance and platelet dysfunction. However, the mechanism through which mutations in *SLFN14* induce enhanced platelet turnover and abnormal platelet function is unknown. Similarly, the pathogenic mechanisms of one of the most recently reported causative genes of IT, GNE, are unknown. Mutations of *GNE*, the gene encoding Glucosamine (UDP-NAcetyl)-2-Epimerase/N-Acetylmannosamine kinase, cause sialuria and hereditary inclusion body myopathy [63] but are also associated with severe thrombocytopenia characterized by shortened platelet lifespan, but the exact mechanisms have not been clarified [48].

## 4. Diagnostic Approach

### 4.1. Introduction

Patients referred for investigation of bleeding symptoms should undergo preliminary laboratory investigations including full blood count, prothrombin time, activated partial thromboplastin time and von Willebrand factor (VWF) screening tests (VWF antigen, ristocetin cofactor activity and factor VIII coagulant activity). If from full blood count thrombocytopenia is identified, a diagnostic work-up for IT should be pursued. If these are normal the presence of an inherited platelet function disorder (IPFD) should be explored. IPFD are listed under Table 2.

The diagnostic approach to ITs can be divided into two steps. The first is the recognition of the hereditary nature of thrombocytopenia, the second is the diagnosis of a specific disorder. In fact, ITs are often confused with acquired thrombocytopenias, leading many patients to receive futile and often dangerous treatments. Careful medical history and accurate evaluation of some simple laboratory parameters help to avoid misdiagnosis [64,65]. A diagnostic algorithm for inherited thrombocytopenias was proposed several years ago and it is still valid to orient towards specific disorders [66,67]. History and clinical examination are crucial for patients with syndromic forms, whereas cell counting and the examination of peripheral blood films may guide diagnosis in non-syndromic forms [68]. However, in most cases genetic studies are required to confirm the diagnostic suspicion [3,69]. Here we propose a diagnostic flow chart for diagnosis of IT.

### 4.2. Clinical Examination

The first step for IT diagnosis is a careful clinical evaluation of the proband, including the personal and family bleeding history. Treatment with drugs (continuous or intermittent), recent infection, previously diagnosed haematologic disease, nonhaematologic diseases known to decrease platelet counts (e.g., eclampsia, sepsis, DIC, anaphylactic shock, hypothermia, massive transfusions), recent live virus vaccination, poor nutritional status, pregnancy, recent organ transplantation from a donor sensitized to platelet alloantigens and recent transfusion of a platelet-containing product in an allosensitized recipient should be excluded. Thrombocytopenia and/or bleeding history in other family members support the hypothesis of an IT, however a negative family history does not exclude it because some forms are recessive or derive from de novo mutations.

The most severe ITs, such as congenital amegakaryocytic thrombocytopenia or biallelic BSS, are typically identified early in infancy because of bleeding diathesis, while for several ITs spontaneous bleeding is absent or very mild explaining why they are often recognized in adult life.

Besides hemorrhagic manifestations, physical examination should also explore other organs/systems abnormalities for syndromic ITs.

In most syndromic forms the associated manifestations are present since the first months of life, such as in CAMT, Jacobsen and Wiskott–Aldrich syndrome and thrombocytopenia with absent radii, while in others they may become apparent later in life, such as renal failure in MYH9-RD, and in the latter case their genetic origin may be missed. 

### 4.3. Laboratory Tests

At the first identification of thrombocytopenia, “pseudothrombocytopenia”, a relatively common artifactual phenomenon caused by platelet clumping in the test tube due to the presence of EDTA (ethylenediaminetetraacetic acid) as anticoagulant accounting for 0.07% to 0.27% of all cases of isolated thrombocytopenia, should be excluded [70].

Evaluation of peripheral blood smears can guide the diagnostic workup because 29 of the 41 forms that have been identified so far display morphological abnormalities of platelets, granulocytes, and/or erythrocytes [68].

When platelet size is reduced, X-linked thrombocytopenia (XLT), WAS and ITs associated with variants in FYB and PTPRJ should be considered [71]. When platelet size is enhanced MYH9-RD, BSS, GPS, thrombocytopenia linked to DIAPH1, FLNA, GATA-1, GNE, TUBB-1, GFI1b, PRKACG, SLF14, TRPM7, TPM4 and ACTN1, Paris-Trousseau thrombocytopenia, PT-VWD, ITGA2B/ITGB3-RT or thrombocytopenia associated with sitosterolemia should be considered. Among these, giant platelets characterize MYH9-RD, bBSS and TUBB1-RT. ITs associated with a normal platelet size instead are ATRUS, SRC-RT, TAR, thrombocytopenia and erythrokeraderma, CYCS-RT, FLI1-RT, IKZF5-RT, THPO-RT, ANKRD26-RT, CAMT, ETV6-RT and FPD/AML. 

Abnormality of platelet granules may be observed in some ITs, with reduced or absent granules with enlarged platelets in GPS and GFI1b-RT and with reduced granules with normal-sized platelets in ANKRD26-RT [13,23,72].

Immunofluorescence performed on blood smears has recently been proposed as a method to identify defective membrane protein expression, disturbed distribution of cytoskeletal proteins, and reduction of α or delta granules, however this method requires interlaboratory validation [68].

Classic tests of platelet function, such as aggregometry (light transmission or impedance aggregometry), flow cytometry, secretion assays, electron microscopy and western blotting, may help for some ITs as subsequent steps in the diagnostic algorithm (Table 3) [18,73,74,75].

The platelet aggregation pattern may be typical of some ITs like biallelic BSS, associated with no response to ristocetin but normal aggregation to all other agonists, or PT-VWD, with increased response to ristocetin.

Measurement of platelet glycoproteins by flow cytometry, using a well-defined set of antibodies, is the gold standard for the diagnosis of biallelic and monoallelic BSS, *ITGA2B/ITGB3*-RT and *GFI1B*-RT.

The measurement of platelet granule content and secretion can reveal alterations, e.g., in WAS and thrombocytopenia with absent radii (TAR) a reduced number of dense-granules has been reported, GPS is characterized by absent or reduced α-granules [78], Paris-Trousseau (PTS) and Jacobsen syndromes show abnormally large α-granules, while patients with *FLNA*-RT show some platelets having a reduced number of α-granules and others with enlarged α-granules [15,79].

Other structural abnormalities, like membranous inclusions, platelet organelle abnormalities, endoplasmic reticulum (ER)-derived inclusion bodies or particulate cytoplasmic structures with immunoreactivity for polyubiquitinated proteins and proteasome (PaCSs) [32], can be detected by electron microscopy in platelets from some specific ITs (Table 3) [18]. 

Additional tests may be required for complex cases, including the measurement of platelet phosphatidylserine expression, by flow cytometry, to detect enhanced procoagulant activity (Stormorken syndrome), spreading or adhesion assays to detect increased spreading in *FLNA*-RT, or western blotting for detection of specific proteins usually absent from platelets (e.g., MYH10 in FPD/AML and FLI1-RT).

Some additional non platelet-related laboratory tests may complement physical examination in the search for syndromic manifestations, like urinalysis, to detect proteinuria as the first sign of renal impairment in *MYH9*-RD, or the liver enzymes, which are elevated in approximately 50% of patients with this disease [80].

### 4.4. Genetic Analysis

While genotyping has mainly been used as a confirmatory test in the past, it is now playing an increasing role in the initial diagnostic approach to IT.

**Figure 2:**
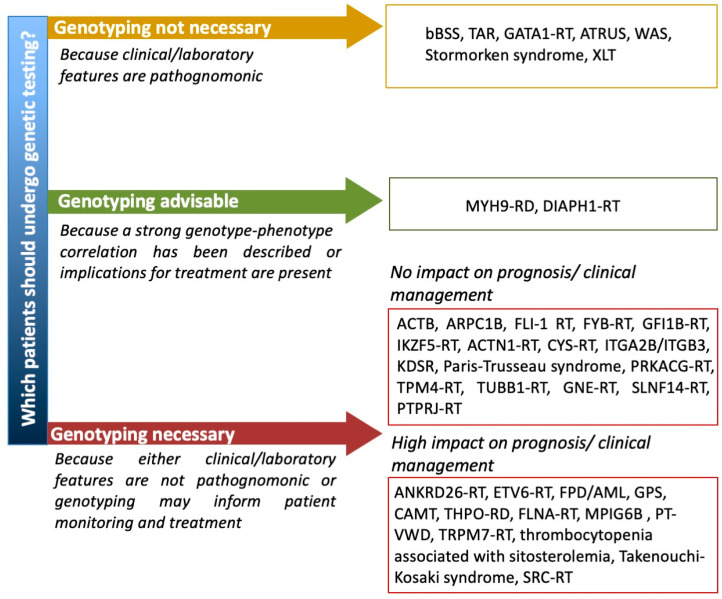
Proposal of a flow chart guiding the use of genetic testing for patients with suspected IT. ACTB = Baraitser–Winter syndrome 1 with macrothrombocytopenia, ARPC1B = Platelet abnormalities with eosinophilia and immune-mediated inflammatory disease, ATRUS = amegakaryocytic thrombocytopenia with radio-ulnar synostosis, bBSS = biallelic Bernard Soulier syndrome, CAMT = congenital amegakaryocytic thrombocytopenia, MPIG6B = thrombocytopenia, anemia and myelofibrosis, PT-VWD = platelet-type von Willebrand disease, RD = related disorder, RT = related thrombocytopenia, TAR = thrombocytopenia with absent radii, XLT = X-linked thrombocytopenia, WAS = Wiskott–Aldrich syndrome.

Until a few years ago, in fact, when the inherited nature of thrombocytopenia was suspected, a series of laboratory tests (e.g., flow cytometry for platelet surface GPs, examination of peripheral blood smear and immunofluorescence assay for MYH9 protein aggregates in neutrophils, platelet aggregometry) were performed to orient towards the candidate gene/genes to be sequenced by Sanger sequencing [66]. The application of high throughput sequencing (HTS) techniques to platelet disorders has allowed for the discovery of several novel genes associated with IT in a few years and has opened the possibility of approaching IT diagnosis by a single-step strategy. In fact, the simultaneous screening of several genes by targeted sequencing platforms, whole exome sequencing (WES) or whole genome sequencing (WGS) has been shown to provide diagnosis in 30% to 50% of patients with suspected IT [81,82,83]. Indeed, HTS is being proposed as a first line diagnostic investigation by an increasing number of authors [82,83,84,85]. However, the interpretation of genetic variants is challenging and requires a careful expert team evaluation in light of a well characterized patient phenotype [84] and when new variants in diagnostic-grade (TIER1) genes are found by targeted sequencing, WES or WGS or new genes are identified by WES or WGS it is essential that rigorous guidelines (i.e., the ACMG guidelines [86]) are applied to confirm their pathogenicity [84]. No guidelines are available yet regarding which suspected IT patients should undergo genetic testing. Some ITs with pathognonomic laboratory or clinical features, such as BSS, TAR, *GATA1*-RD, ATRUS, Stormorken syndrome and WAS, can be clearly diagnosed without the need of genetic testing. For other ITs, for which a strong genotype–phenotype correlation has been described, e.g., *MYH9*-RD, genotyping may be advisable for prognostic evaluation and possible preventive intervention. Other forms that do not have any specific diagnostic, clinical or laboratory features would require genetic testing for definite diagnosis. However, for some of these, e.g., *ACTN1*-RT or *TUBB1*-RT, a genetic diagnosis does not have any significant impact on patient management, while for others it may inform patients monitoring and treatment. Among these there are thrombocytopenias with normal platelet volume, including forms like FPD/AML, *ANKRD26*-RT and *ETV6*-RT which are predisposed to hematological malignancies (Figure 2). There are ethical implications of detecting variants in these genes and other unexpected genetic defects, such as a carrier status of a recessive gene. It is thus recommended to strictly follow an informed consent protocol ensuring that patients comprehend the possible implications of unsolicited genetic findings [85].

In summary, the optimal diagnostic approach to ITs is still being debated and a combination of clinical/traditional laboratory approach with advanced gene sequencing techniques may provide the highest rate of diagnostic success [69], and the best patient management.

### 4.5. Undefined Aspects and Possible Future Research Lines

A consensus on the classification of ITs has not been reached yet, but it would be highly advisable to avoid, for example, ambiguity on disease nomenclature.

A guidance flow chart about which suspected IT patients should undergo genetic testing is not yet available and the generation of consensus documents promoted by the relevant international scientific societies (ISTH, EHA, ASH) is highly warranted.

Moreover, development of guidelines on informed consent documents, reporting of new variants in variant databases to improve variant classification, development of user-friendly interpretation softwares of HTS results, promotion of research for discovery of new genes causing IT and development of advanced cell-based models to study platelet formation and function are valuable future perspectives.

## 5. Bleeding and Other Manifestations

Bleeding manifestations of IT are of variable severity, ranging from severe in rare cases, recognized within a few weeks from birth, to mild or absent [87]. They are characterized by mucocutaneous symptoms, including epistaxis, easy bruising, petechiae, prolonged bleeding from cuts, gum bleeding, hematuria and menorrhagia in women but also by excessive bleeding after surgery or post-partum hemorrhages [5,88,89].

A recent large systematic investigation on the diagnostic utility of the ISTH bleeding assessment tool (ISTH-BAT) in patients with inherited platelet disorders showed that the bleeding history of most patients with IT without associated platelet function defect is not severe, with a median ISTH-BAT bleeding score (BS) of 2, quite comparable to that of healthy subjects [65]. Usually, the bleeding risk is negligible in subjects with more than 100 × 10^9^ platelets/L, mild/moderate in subjects with 50–100 × 10^9^ platelets/L (risk of hemorrhages on the occasion of major hemostatic challenges) and significant when platelets are lower than 50 × 10^9^/L, especially when below 20 × 10^9^/L [90].

On the other hand, in ITs associated with defective platelet function (Table 2) the bleeding history shows a moderate/severe hemorrhagic tendency, with high BS, like in patients with biallelic-BSS (median BS 8.5), GPS (median BS 12) and ITGA2B/ITGB3-RT (median BS 8) [65]. For other rare and/or underdiagnosed ITs, such as PT-VWD, GATA-1 RD or CYCS-RT [65,91], the hemorrhagic risk is still poorly defined.

Pregnancy and delivery are a major concern for patients with ITs because both the mother and the affected newborn may be at risk of bleeding. A large multicentric, retrospective study evaluated 339 pregnancies in 181 women with 13 different forms of IT and showed that neither the degree of thrombocytopenia nor the severity of the bleeding tendency worsened during pregnancy and that, in general, the course of pregnancy did not differ from that of healthy subjects. However, post-partum hemorrhage was more frequent in ITs, ranging from 6.8% to 14.2% vs. 3% to 7% in control women, with the degree of thrombocytopenia (platelet count at delivery below 50 × 10^9^/L) and previous history of severe bleeding being predictive of delivery-associated hemorrhage. Patients with MYH9-RD, ANKRD26-RT, biallelic and monoallelic BSS, FPD/AML, GPS and PT-VWD showed the highest frequency of post-partum bleeding [72].

Delivery-related neonatal hemorrhages were instead quite rare (4.5% of affected newborns), although two fatal cerebral hemorrhages out of 278 childbirths were reported [72]. Recently, pregnancy and delivery in a woman with DIAPH1-RD were reported with no changes in platelet count and no bleeding at delivery or postpartum [92].

Another feared complication in patients with IT is excessive bleeding after surgery and a multicentric, retrospective worldwide study recently assessed the bleeding complications of surgery, the preventive and therapeutic approaches adopted and their efficacy in patients with inherited platelet disorders. The study showed that the frequency of surgical bleeding was higher in patients than in healthy controls (19.7% vs. 1.4%–6%), however in patients with IT and normal platelet function bleeding incidence was relatively low (13.4%) with 68 × 10^9^/L platelets being the threshold below which bleeding rate increased significantly, while in IT patients with associated platelet dysfunction post-surgical hemorrhage was frequent, in particular in those with bBSS (44.4%), FPD/AML (30.8%), GPS (23.5%) and ITGA2B/ITGB3-RT (22.7%) [93].

Although bleeding is conventionally considered the main clinical complication in patients with IT, some ITs have the propensity to develop other disorders, including hematological malignancies or bone marrow aplasia [82,94], while others have associated syndromic manifestations, like skeletal malformations, liver and kidney malfunction and deafness. For instance, 40% of subjects with FDP/AML develop acute myelogenous leukemia (AML) or myelodysplastic syndromes (MDS) with a median age of onset of 33 years old, 8% of subjects with *ANRD26*-RT develop MDS or AML and 25% of subjects with ETV6-RT develop hematologic malignancies [95]. Moreover, genotype–phenotype correlation studies in MYH9-RD patients have reported that variants in the head domain of MYH9 are associated with more severe thrombocytopenia and a higher frequency and/or rapid progression of deafness and nephropathy than variants in the tail domain, with the amino acid substitution p.Arg702Cys being associated with the most severe phenotype [96,97].

## 6. Prophylaxis and Treatment Options

The management of patients with IT should aim to prevent bleeding and treat hemorrhages but also to arrest or slowdown the development of systemic complications or treat them [98,99].

### 6.1. General Prophylactic Measures

Patients should avoid drugs interfering with platelet function, such as aspirin and non-steroidal anti-inflammatory drugs, perform accurate dental hygiene, and, for the most serious forms, avoid contact sports. Given the possibility that these patients will be exposed to blood transfusions during their life, it is important that they receive immunization against hepatitis A and B and annual liver function tests [99]. Correction of iron deficiency is often required, especially in children and young women [100]. Prenatal diagnosis can be carried out for the most serious forms when the familial mutation is known. Moreover, mutational screening of potential sibling donors is highly warranted for patients with risk of developing hematological malignancy who may therefore require future hematopoietic stem cells transplantation, such as in FPD/AML, ANKRD26-RT and ETV6-RD [101]. Screening for renal failure and cataract in MYH9-RT, for deafness in MYH9-RD and DIAPH1-RT, and for myelofibrosis and immune disorders in GPS is warranted. In fact, immune derangement in GPS has been recently shown, with more than one-half of patients having detectable autoantibodies and one-quarter clinically evident autoimmune disorders, including Hashimoto’s thyroiditis, rheumatoid arthritis, alopecia, discoid lupus erythematosus, vitiligo and atypical autoimmune lymphoproliferative syndrome usually associated with cytopenia of at least one leukocyte type [79].

### 6.2. Female Hormones

Menarche, particularly in patients with BSS, may be associated with excessive bleeding [89]. This can be treated by intravenous (IV) infusion of high-dose conjugated estrogen for 24–48 h followed by high doses of oral estrogen–progestin. Thereafter, a combined oral contraceptive can be given continuously for 2–3 months. In women in whom antifibrinolytic agents fail to decrease menorrhagia, long term oral contraceptives can be given, especially when iron deficiency anemia develops [102].

### 6.3. Local Hemostatic Measures

Electrocautery and nasal packing are used for epistaxis, while compression, suturing, and application of gelatin sponges or gauzes soaked in tranexamic acid for accidental or surgical wound bleedings. Mouthwashes with tranexamic acid, application of fibrin sealant or absorbable gelatin sponge with topical thrombin may be useful for gingival bleeding. Non-conventional hemostatic agents, such as Ankaferd Blood Stopper has been used in some patients in cases of inefficacy of the classical measures [103]. A relatively new method proposed for the acceleration of wound healing are platelet-rich clots, due to the release of several growth factors from platelets, although it still requires standardization and validation [104]. Autologous platelet-rich clots were used with success, in conjunction with tranexamic acid given orally, to prevent bleeding during dental extraction for a patient with PT-VWD [105].

### 6.4. Platelet Transfusions

Given the risk of alloimmunization, allergic reactions and infections, platelet transfusion should be used only for severe bleeding which cannot be managed by local measures. Moreover, to prevent HLA-alloimmunization and reactions, HLA-matched and leukodepleted concentrates should be used. In case alloimmune antibodies develop, e.g., against GPIb/IX/V in BSS, immunosuppression and/or plasmapheresis can restore platelet transfusion efficacy.

Of note, the use of pre-operative antihemorrhagic prophylaxis was associated with a lower bleeding frequency in patients with inherited platelet function disorders but not with IT, indeed in the latter group bleeding was reported in 12.7% of the procedures carried out without preparation and in 14.9% of the procedures carried out with pre-operative antihemorrhagic prophylaxis. On the other hand, the choice of the preventive measures did not appear to be always appropriate, in fact platelet transfusions, the most frequently used prophylactic treatment, revealed to be poorly effective, suggesting that either other treatments are required, or that the way platelet transfusions are employed (amount, type, timing) is inappropriate [93].

### 6.5. Antifibrinolytic Agents

Antifibrinolytic agents (AF), such as ε-aminocaproic acid or tranexamic acid, used as single drugs or in association with other treatments, have been shown to be useful for covering minor surgery in patients with IT or in arresting epistaxis, gingival bleeding or menorrhagia [93,96]. However, no specific prospective clinical study on the effectiveness of these drugs in ITs has been performed, and should therefore be considered empirical. AFs are usually contraindicated for hematuria given the risk of clot formation in the urinary tract [106], however exceptions have been reported [107] and the evidence of AF-associated clot risk is weak and based on old, uncontrolled data [108].

### 6.6. Desmopressin

Desmopressin (1-deamino-8-D-arginine vasopressin, DDAVP) is an approved treatment for mild hemophilia A and type 1 von Willebrand disease, but is also used for congenital and acquired defects of platelet function because it has a general prohemostatic effect and it enhances the procoagulant activity of platelets [109]. Clinical studies on the efficacy of DDAVP in ITs are lacking, however DDAVP has been shown to successfully cover minor surgery in some IT patients, such as MYH9-RD, ANKRD26-RT and Paris-Trousseau syndrome [92,110,111]. In elderly patients and in patients with a history of cardiovascular disease DDAVP should be used with caution for increased risk of thrombosis as well as in infants below two years of age for the risk of fluid retention.

### 6.7. VWF-Rich Concentrates

VWF-rich concentrates are the most effective treatments, together with platelet transfusions, for major bleeding in PT-VWD. The dose of VWF-rich concentrates depends on the level of VWF:RCo and can be adjusted on demand. A target of 50–60% VWF:RCo/VWF activity in major surgery and 30–50% in minor ones is advisable (typically from 10 to 30 U/kg at 12 h intervals) [76].

### 6.8. Activated Recombinant Factor VIIa (rFVIIa)

rFVIIa is currently approved for treating hemophiliacs with inhibitors and patients with Glanzmann thrombasthenia [112]. In the setting of ITs, rFVIIa has been successfully used as prophylactic measure for invasive procedures in biallelic BSS, PT-VWD and TAR [113]. Severe adverse events, including myocardial infarction, ischemic stroke and venous thromboembolism have occasionally been reported.

### 6.9. Eltrombopag

Eltrombopag is an oral TPO-mimetic indicated for chronic refractory immune thrombocytopenic purpura (ITP), severe aplastic anemia and HCV-related thrombocytopenia. It has been shown to be effective in increasing transiently the platelet count in ITs. In two phase 2 clinical trials, eltrombopag given for three to six weeks was shown to be safe and effective in increasing platelet count and reducing bleeding symptoms in 10 out of 11 patients with *MYH9*-RD [114] and in 21 out of 23 patients with *MYH9*-RD, *ANKRD26*-RD, XLT/WAS, monoallelic BSS and *ITGA2B/ITGB3*-RT [115].

Long-term eltrombopag (i.e., eltrombopag administration for more than six months) to maintain stable safe platelet counts has been used in eight patients with WAS/XLT and severe thrombocytopenia. Five responded well, obtaining a stable increase of platelet count and reduction of spontaneous bleeding without major adverse events [116]. However, potential side effects of a life-long treatment (such as bone marrow fibrosis) need to be carefully considered.

Moreover, eltrombopag has been successfully used for preparation to surgery in patients with MYH9-RD and severe thrombocytopenia [117,118,119]. Recently, treatment with eltrombopag allowed to attain a safe and stable platelet count to allow chemotherapy in a patient with MHY9-related disorder and pancreatic cancer and permitted to perform endoscopic placement of a biliary stent with no bleeding complications [120].

### 6.10. Hematopoietic Stem Cell Transplantation (HSCT) and Gene Therapy

HSCT has become the treatment of choice for patients with WAS, with a 5-year overall survival rate of 90%–100% [121], and with CAMT, with a long-term survival rate of 80% [122], patients who have, without this treatment, a life expectancy of 15 years and a few months, respectively [122,123]. Bone marrow transplantation from HLA-identical donors has also been used with success in some cases of BSS with severe hemorrhage and/or alloantibodies [124] and in patients with XLT [125]. However, a careful evaluation of the risk-benefit ratio must always be made.

In humans, the feasibility of gene therapy has been proven in patients with WAS with sustained clinical benefit, normalization of platelet volume and partial increase of platelet count [126,127,128]. Research is ongoing for gene therapy of BSS in a mouse model [129] and of CAMT in induced pluripotent stem cells [22].

### 6.11. Splenectomy

Splenectomy is effective in patients with WAS/XLT, increasing platelet count and reducing the incidence of serious bleedings, however it significantly increases the incidence of subsequent severe infectious events and it does not increase overall survival [130]. Thus, the risk–benefit balance should be carefully weighed in each patient and vaccination and anti-infective prophylaxis should always be performed [131].

## 7. Conclusions

Thrombocytopenia is a frequent condition for the internist and the hematologist, and its differential diagnosis is frequently complex and cumbersome. Among the various possible etiologies of thrombocytopenias, inherited forms should be promptly recognized to avoid unnecessary, and frequently potentially dangerous treatments, and to allow the precise formulation of prognostic expectations.

Recent advances in the understanding of the pathogenic mechanisms of gene variants provoking thrombocytopenia, of the phenotypic manifestations of specific IT-associated gene variants, in the diagnostic approach to ITs and in the therapeutic opportunities have yielded improvements in patient care and deeper insight into the physiologic regulation of circulating platelet levels.

Future challenges are the identification of the genetic cause of the remaining 50% of so far unclassified ITs, the unraveling of the precise phenotypic features of several IT forms, the understanding of the role in megakaryopoiesis of some mutated genes found to be associated with ITs, the development of new therapeutic approaches and of the best use of those currently available and the identification of sophisticated in vitro models of megakaryopoiesis to allow better modelling studies with patient derived MK or induced pluripotent stem cells.

Only continued research and the creation of a wide international collaborative network among investigators and clinicians in the field will allow to respond to these.

## Figures and Tables

**Table 1:** IT classified based on the defective step of platelet count regulation involved.

Defective Step of Thrombopoiesis	Affected Gene	Disorder	Pathogenic Mechanism (Reference)	Additional Features (e.g., Syndromic Manifestations, Predisposition)
Defective megakaryocyte maturation	ANKRD26	ANKRD26-related thrombocytopenia	Loss of ANKRD26 silencing during the last phases of megakaryocytopoiesis causes ERK1/2 phosphorylation that interferes with megakaryocyte maturation [13]	Predisposition to hematological malignancies
	ETV6	ETV6-related thrombocytopenia	ETV6 is a transcriptional repressor that promotes the late phases of megakaryopoiesis. Mutations in ETV6 cause defective megakaryocyte maturation and impaired proplatelet formation [14]	Predisposition to hematological malignancies
	FLI1	FLI1-related thrombocytopenia	FLI1 is a transcription factor regulating many genes associated with megakaryocyte development. Therefore, FLI1 mutations promote defective megakaryocyte maturation [15]	Not reported
	FLI1 deletion	Paris-Trousseau syndrome/Jacobsen syndrome		Abnormalities of heart and face, intellectual disabilities
	FYB	FYB-related thrombocytopenia	ADAP is a protein involved in the remodeling of cytoskeleton. Mutations in ADAP cause defective maturation of megakaryocytes and clearance of platelets [16]	Mild iron deficiency anemia
	GATA1	GATA1-relate disease	GATA1 is a transcription factor regulating many genes associated with megakaryocyte development therefore GATA1 defects cause alterations of megakaryocyte maturation [17]	Dyserythropoietic anemia, beta-thalassemia, congenital erythropoietic porphyria, splenomegaly
	GFI1B	GFI1B-related thrombocytopenia	GFI1B is a transcription factor involved in homeostasis of hematopoietic stem cells and development of megakaryocytes therefore GFI1B defects cause alterations of megakaryocyte maturation [18]	Mild myelofibrosis
	HOXA11	Amegakaryocytic thrombocytopenia with radio-ulnar synostosis	HOXA11 is a transcription factor involved in the regulation of early hematopoiesis, its defect causes reduced number of megakaryocytes [19]	Bilateral radioulnar synostosis, severe bone marrow failure culminating in aplastic anemia in majority of cases, cardiac and renal malformations, hearing loss, clinodactyly, skeletal abnormalities, pancytopenia
	MECOM		MECOM is a transcription factor involved in the regulation of early hematopoiesis, its defect causes reduced number of megakaryocytes [20]	
	IKZF5	IKZF5-related thrombocytopenia	IKZF5 is a previously unknown transcriptional regulator of megakaryopoiesis [21]	Not reported
	MPL	Congenital amegakaryocytic thrombocytopenia	MPL is the receptor for thrombopoietin. MPL defects cause impaired thrombopoietin binding and thus impaired megakaryocyte maturation [22]	Acquired bone marrow aplasia
	NBEAL2	Gray platelet syndrome	Mutations in NBEAL2 cause impaired megakaryocyte maturation however its role in megakaryocytopoiesis is not clear [23]	Myelofibrosis, immune dysregulation (autoimmune diseases, positive autoantibodies, reduced leukocyte counts), proinflammatory profile
	RBM8A	Thrombocytopenia-absent radius	RBM8A is a protein of the exon-junction complex involved in RNA processing. It has been hypothesized that RBM8A defects cause wrong mRNA processing of unknown components of the TPO-MPL pathway impairing megakaryocyte maturation [24]	Bilateral radial aplasia, anemia, skeletal, urogenital, kidney, heart defects
	RUNX1	Familial platelet disorder with predisposition to hematological malignancies	RUNX1 is a transcription factor regulating many genes associated with megakaryocyte development therefore RUNX1 mutations promote defective megakaryocyte maturation [25]	Predisposition to hematological malignancies
	THPO	THPO-related disease	THPO is the gene for thrombopoietin, essential for hematopoietic stem cell survival and megakaryocyte maturation [26]	Bone marrow aplasia
Defective platelet production/increased clearance	ACTB	Baraitser–Winter syndrome 1 with macrothrombocytopenia	Mutations in β-cytoplasmic actin inhibit the final stages of platelet maturation by compromising microtubule organization [27]	Microcephaly, facial anomalies, mild intellectual disability, developmental delay
	ACTN1	ACTN1-related thrombocytopenia	ACTN-1 is involved in cytoskeletal remodeling, defects in ACTN-1 cause defective proplatelet formation [28]	Not reported
	ARPC1B	Platelet abnormalities with eosinophilia and immune-mediated inflammatory disease	The actin-related protein 2/3 complex (Arp2/3) is a regulator of the actin cytoskeleton and its mutation causes impaired proplatelet formation [29]	Immunodeficiency, systemic inflammation, vasculitis, inflammatory colitis, eosinophilia, eczema, lymphadenomegaly, hepato-splenomegaly, growth failure
	CYCS	CYCS-related thrombocytopenia	CYCS is a mitochondrial protein with a role in respiration and apoptosis. Mutations in CYCS cause ectopic premature proplatelet formation with an unknown mechanism [30]	Not reported
	DIAPH1	DIAPH1-related thrombocytopenia	DIAPH1 is involved in cytoskeletal remodeling, defects in DIAPH1 cause defective proplatelet formation [31]	Hearing loss
	FLNA	FLNA-related thrombocytopenia	Filamin A is involved in cytoskeletal remodeling, defects in FLNA cause defective proplatelet formation [32]	Periventricular nodular heterotopia and otopalatodigital syndrome spectrum of disorders
	GP1BA, GP1BB, GP9 (loss of function)	Bernard–Soulier syndrome monoallelic	The intracellular portion of the GPIb/IX/V complex links the receptor to the cytoskeleton. Disruption of this link causes impaired proplatelet formation [33]	Not reported
		Bernard–Soulier syndrome biallelic		
	GP1BA (gain of function)	Platelet-type von Willebrand disease	The extracellular portion of the GPIb/IX/V complex binds VWF. Constitutive binding of VWF to its receptor triggers the Src kinases pathway causing impaired proplatelet formation, ectopic platelet production and increased platelet clearance [34]	Not reported
	ITGA2B, ITGB3	ITGA2B/ITGB3-related thrombocytopenia	Constitutive activation of α_IIb_β_3_ causes cytoskeletal perturbation leading to impaired proplatelet formation [35,36]	Not reported
	KDSR	Thrombocytopenia and erythrokeraderma	KDSR is an essential enzyme for de novo sphingolipid synthesis, this suggests an important role for sphingolipids as regulators of cytoskeletal organization during megakaryopoiesis and proplatelet formation [37]	Dermatologic involvement ranging from hyperkeratosis/ erythema to ichthyosis. One family with no or very mild skin lesions but associated anemia has been reported
	MYH9	MYH9-related disorder	MYH9 regulates cytoskeleton remodeling and mediates signal transduction pathways involved in proplatelet formation. Abnormalities of MYH9 cause hyperactivation of the Rho/ROCK pathway causing ectopic platelet formation [38]	Kidney disease, cataract, deafness, elevated liver enzymes
	MPIG6B	Thrombocytopenia, anemia and myelofibrosis	G6b-B is a transmembrane receptor with an ITIM motif with a not well defined role in proplatelet formation [39]	Microcitic anemia, myelofibrosis, leukocytosis may be present
	PRKACG	PRKACG-related thrombocytopenia	PKA activates many proteins involved in megakaryocyte and platelet function, among them FLNa and GPIbβ therefore its dysfunction causes impaired proplatelet formation [40]	Not reported
	STIM1	Stormorken syndrome	STIM1 mutations cause a constitutively active store operated Ca^2+^ release-activated Ca^2+^ (CRAC) channel which triggers Ca^2+^ entry with consequent increased clearance of activated platelets [41]	Tubular myopathy and congenital myosis. Severe immune dysfunction
	TRPM7	TRPM7-related thrombocytopenia	Defects of the Mg^2+^ channel TRPM7, a regulator of embryonic development and cell survival, cause cytoskeletal alterations resulting in impaired proplatelet formation [42]	Atrial fibrillation
	TPM4	TPM4-related thrombocytopenia	Tropomyosin 4 is an actin cytoskeletal regulator. Insufficient TPM4 expression in human and mouse megakaryocytes resulted in a defect in the terminal stages of platelet production [43]	Not reported
	TUBB1	TUBB1-related thrombocytopenia	Tubulin beta1 is a major component of microtubules therefore defects in TUBB1 cause impaired proplatelet formation [44]	Not reported
	WAS	Wiskott–Aldrich syndrome	The WASP protein is a regulator of the actin cytoskeleton and its defect causes ectopic platelet formation and increased platelet clearance [45]	Immunodeficiency, hematopoietic malignancies, eczema, autoimmune hemolytic anemia.
		X-linked thrombocytopenia		Not reported
Other/unknown pathogenic mechanism	ABCG5, ABCG8	Thrombocytopenia associated with sitosterolemia	ABCG5 and ABCG8 regulate plant sterol and cholesterol absorption. It is supposed that sterol-enriched platelets are more rapidly cleared [46]	Xanthomas and pre-mature coronary atherosclerosis due to hypercholesterolemia
	CDC42	Takenouchi-Kosaki syndrome with macrothrombocytopenia	CDC42 is a critical molecule in various biological processes including the cell cycle, cell division, and the formation of the actin cytoskeleton [47]	Defective growth and psychomotor development, intellectual disability, facial abnormalities, brain malformation, muscle tone abnormalities, immunodeficiency, eczema, hearing/visual disability, lymphedema, cardiac, genitourinary, and/or skeletal malformations
	GNE	GNE-related thrombocytopenia	GNE encodes an enzyme involved in the sialic acid biosynthesis pathway and it is known that thrombocytopenia is associated with increased platelet desialylation [48]	Some patients presented myopathy with rimmed vacuoles with onset in early adulthood
	SLNF14	SLNF14-related thrombocytopenia	SLNF14 is an endoribonuclease and its role in the generation of thrombocytopenia is unknown [49]	Not reported
	SRC	SRC-related thrombocytopenia	Src-family kinase regulates multiple signaling pathways, its role in the generation of thrombocytopenia is unknown [50]	Myelofibrosis, bone pathologies, bone marrow dysplasia, splenomegaly, congenital facial dysmorphism
	PTPRJ	PTPRJ-related thrombocytopenia	PTPRJ is a protein tyrosine phosphatase expressed abundantly in platelets and megakaryocytes, its role in the generation of thrombocytopenia is unknown [51]	None

**Table 2:** Inherited platelet function disorders: disorders in which platelet dysfunction is the dominant phenotypic feature independent of platelet count.

Disease	Inheritance	Gene	Bleeding Diathesis
Arthrogryposis, renal dysfunction and cholestasis	AR	*VPS33B* *VIPAS39*	Severe
CalDAG-GEFI related platelet disorder	AR	*RASGRP2*	Moderate-severe
Cediak-Higashi Syndrome	AR	*CHS1*	Moderate-severe
Combined alpha-delta granule deficiency	AR/AD	Unknown	Mild-moderate
COX-1 deficiency	AR/AD	PTGSA	Moderate-severe
Delta granule deficiency	AR/AD	Unknown	Mild-moderate
Glanzmann thrombasthenia	AR	*ITGA2B*, *ITGB3*	Moderate-severe
Glycoprotein IV (GPIV) deficiency	AR	*GP4*	Mild
Glycoprotein VI (GPVI) deficiency	AR	*GP6*	Mild
G_s_ platelet defect	AD (if paternally inherited)	*GNAS*	Mild
Hermansky–Pudlak syndrome	AR	*HPS1*, *ADTB3A*, *HPS3*, *HPS4*, *HPS5*, *HPS6*, *DTNBP1*, *BLOC1S3*, *AP3D1*, *BLOC1S6*	Moderate-severe
Leukocyte adhesion deficiency, type III	AR	*FERMT3*	Moderate-severe
P2Y12 deficiency	AR	*P2RY12*	Moderate-severe
Phospholipase A_2_ (cPLA_2_) deficiency	not determined	*PLA2G4A*	Moderate-severe
PKCδ deficiency	AR	*PRKCD*	Absent
Primary secretion defect	AR/AD	Unknown	Mild-moderate
Quebec platelet disorder	AD	*PLAU*	Moderate-severe
Scott syndrome	AR	*TMEM16F*	Mild-moderate
Thromboxane A2 receptor defect	AD	*TBXA2R*	Mild
T_x_ synthase deficiency	AD/AR	*TBXAS1*	Moderate

**Table 3:** Main features of inherited thrombocytopenias.

Form	Disease	Inheritance	Degree of Thrombocytopenia	Key Laboratory Features	References
**Syndromic**	Amegakaryocytic thrombocytopenia with radio-ulnar synostosis (ATRUS)	AD	severe	Normal platelet size and morphology	[19,20]
	Baraitser–Winter syndrome 1 with macrothrombocytopenia	AD	absent	Macrothrombocytopenia; leukocytosis with eosinophilia, leukopenia	[27]
	FLNA-related thrombocytopenia	XL	moderate	Macrothrombocytopenia; impaired platelet aggregation GPVI-triggered; heterogeneous α-granules, occasionally giant; abnormal distribution of FLNa	[32]
	GATA-1-related disease	XL	severe	Macrothrombocytopenia; reduced platelet aggregation by collagen and ristocetin; reduced α-granule content and release	[17]
	GNE-related thrombocytopenia	AR	from mild to severe	Macrothrombocytopenia	[48]
	Gray platelet syndrome	AR	moderate/severe	Macrothrombocytopenia; grey or pale platelets; dyserytropoiesis; absence of α-granules; defective TRAP-induced platelet aggregation	[23]
	Paris-Trousseau thrombocytopenia, Jacobsen syndrome	AD	severe	Macrothrombocytopenia; defective platelet aggregation by thrombin; giant α-granules	[15]
	Platelet abnormalities with eosinophilia and immune-mediated inflammatory disease	AR	moderate	Small platelets; eosinophilia; reduced platelet spreading; decreased platelet dense granules	[29]
	PTPRJ-related thrombocytopenia	AR	moderate/severe	Microthrombocytopenia; impaired activation by the GPVI-specific agonist convulxin and the thrombin receptor-activating peptide but normal response to ADP	[51]
	SRC-related thrombocytopenia	AD	moderate/severe	Platelets deficient in granules and rich in vacuoles	[50]
	Stormorken syndrome	AD	moderate/severe	Howell-Jolly bodies in red blood cells; enhanced annexin V binding, defective GPIIb/IIIa activation (PAC-1)	[41]
	Takenouchi-Kosaki syndrome with macrothrombocytopenia	AD	absent	Macrothrombocytopenia, abnormal platelet spreading and filopodia formation	[47]
	Thrombocytopenia-absent radius syndrome (TAR)	AR	severe	Normal platelet size and morphology, thrombocytopenia	[24]
	Thrombocytopenia and erythrokeraderma	AR	moderate	Thrombocytopenia and presence of 3-keto-dihydrosphingosine in plasma	[37]
	Thrombocytopenia, anemia and myelofibrosis	AR	mild/moderate	Macrothrombocytopenia, anemia	[39]
	Wiskott–Aldrich syndrome	XL	severe	Microthrombocytopenia; Reduced α/δ granules release	[45]
	X-linked thrombocytopenia	XL	mild/moderate	Microthrombocytopenia; Reduced α/δ granules release	[45]
**Non-syndromic**	ACTN1-related thrombocytopenia	AD	mild	Macrothrombocytopenia	[28]
	Bernard Soulier syndrome monoallelic biallelic	AD AR	mild moderate/severe	Macrothrombocytopenia; lack of platelet agglutination to ristocetin with normal aggregation to other agonists; severe reduction or complete lack of GPIb/IX/V	[33]
	CYCS-related thrombocytopenia	AD	mild	Normal platelet size and morphology	[30]
	FLI1-related thrombocytopenia	AD/AR	moderate	Reduced platelet aggregation in response to collagen and PAR-1 agonists; δ-granule deficiency	[15]
	FYB-related thrombocytopenia	AR	moderate/severe	Microthrombocytopenia; increased expression of P-selectin and PAC-1 by resting platelets but impaired upon stimulation with ADP	[16]
	GFI1b-related thrombocytopenia	AD/AR	mild/moderate	Macrothrombocytopenia; dyserytropoiesis; reduced α-granule content and release; diminished expression of GPIbα, red cell anisocytosis	[18]
	IKZF5-related thrombocytopenia	AD	absent	Thrombocytopenia; deficiency of platelet alpha granules.	[21]
	ITGA2B/ITGB3-related thrombocytopenia	AD	mild/moderate	Macrothrombocytopenia; reduced GPIIb/IIIa; defective GPIIb/IIIa activation (PAC-1)	[35,36,54]
	PT-VWD	AD	mild/moderate	Macrothrombocytopenia; increased response to ristocetin and decreased VWF-ristocetin cofactor activity (VWF:RCo) Mixing tests discriminate the plasmatic (VWD type2B) from platelet (PT-VWD) origin of hyperreactivity to ristocetin	[36,76,77]
	PRKACG-related thrombocytopenia	AR	severe	Macrothrombocytopenia; defective platelet α_IIb_β_3_ activation and P-selectin exposure in response to TRAP6; defective Ca^2+^ mobilization in response to thrombin	[40]
	THPO-related thrombocytopenia	AD	mild	Normal or slightly increased platelet size	[26]
	TRPM7-related thrombocytopenia	AD	mild/moderate	Macrothrombocytopenia; aberrant distribution of granules	[42]
	Tropomyosin 4 (TPM)-related thrombocytopenia	AD	mild	Macrothrombocytopenia	[43]
	TUBB-1-related thrombocytopenia	AD	mild	Macrothrombocytopenia; platelet anisocytosis	[44]
	SLFN14-related thrombocytopenia	AD	mild/moderate	Macrothrombocytopenia; δ-granule deficiency with decreased ATP secretion in response to ADP, collagen and TRAP-6	[49]
**Forms predisposing to additional diseases**	ANKRD26-related thrombocytopenia	AD	mild/moderate	Reduced α-granules in some patients	[13]
	Congenital amegakaryocytic thrombocytopenia (CAMT)	AR	severe	Elevated serum levels of TPO	[22]
	DIAPH1-related thrombocytopenia	AD	mild/severe	Macrothrombocytopenia	[31]
	ETV6-related thrombocytopenia	AD	mild/moderate	Decreased ability of platelets to spread on fibrinogen covered surfaces; abnormal clot retraction	[14]
	Familial platelet disorder with predisposition to hematological malignancies (FPD/AML)	AD	moderate	Abnormal aggregation in response to multiple agonists; δ (occasionally α)-granule deficiency	[25]
	MYH9-related disease	AD	mild/severe	Macrothrombocytopenia; Döhl-like body cytoplasmic leukocyte inclusions	[38]
	Thrombocytopenia associated with sitosterolemia		moderate/severe	Macrothrombocytopenia; hyperactivatable platelets with constitutive binding of fibrinogen to α_IIb_β_3_ integrin; shedding of GPIbα; impaired platelet adhesion to von Willebrand factor	[46]
